# Palliative and end-of-life care initiatives for people dying from cancer in India: a narrative review

**DOI:** 10.3332/ecancer.2024.1822

**Published:** 2024-12-12

**Authors:** Naveen Salins, Krithika Rao, Anuja Damani, Sushma Bhatnagar, Srinagesh Simha

**Affiliations:** 1 Medicine and Supportive Care, Kasturba Medical College, Manipal Academy of Higher Education, Manipal 576104, Karnataka, India; 2 The Department of Onco-Anaesthesia and Palliative Medicine, Institute Rotary Cancer Hospital, All India Institute of Medical Sciences, Delhi 110029, India; 3 Karunashraya, The Bangalore Hospice Trust, Bengaluru 560037, India

**Keywords:** cancer, palliative care, end-of-life care, initiatives, India

## Abstract

India is facing a growing burden of cancer, resulting in high cancer-associated mortality. However, the rise in cancer incidence is disproportionately high compared to access and provision of palliative care. This review aims to identify gaps in Indian cancer palliative care and recognises initiatives instituted to mitigate them. The narrative review was conducted using the four-step method described by Demiris et al., synthesising both empirical and non-empirical literature. A lack of capacity to provide palliative care was identified as a significant barrier. Initiatives such as setting up palliative care services in cancer treatment centres, improving community palliative care access, structured palliative care training to develop specialists, improving opioid availability and creating policies have been helpful. A significant proportion of people in India experience health-related suffering, and developing a tool to identify this suffering proactively would be beneficial. Several cancer centres are testing integrated cancer palliative care models in various cancer subsites. However, these are preliminary works and are yet to be established. People in India face distress due to high health-related costs, and initiatives like hospices and home-based palliative care services with no cost to patients and families provide significant relief. Caregivers experience a considerable burden while caring for their loved ones with life-limiting illnesses, and they are supported through respite palliative care services offered in some parts of India.

## Background

In 2022, India saw 1.4 million new cancer cases annually, at 100 cases per 100,000 people [1]. A lifetime risk of developing cancer is present in one out of nine individuals in India, and breast cancer is common in women, while lung cancer is common in men [1]. For children, the incidence of cancer ranges from 65 cases per million in girls to 95 cases per million in boys [2].

Cancer incidence has consistently risen, with more than a million new cases reported yearly [3]. It is predicted that by 2040, there will be almost 2 million new cases and over 1 million deaths attributed to cancer in India [3]. From 2000 to 2019, India had 12.85 million deaths caused by 23 major types of cancer. The most common cancers contributing to mortality were those involving the mouth and oropharynx (15.6%), stomach (10.6%), lungs (9.6%), breasts (9%) and colon and rectum (8%) [4]. The cancer mortality rate in India remains high, with less than 30% of patients surviving 5 years or longer after diagnosis [5]. This is mainly because most patients with cancer are diagnosed at advanced stages (3 and 4) due to delayed presentation and limited access to treatment [5]. It, coupled with the high therapy costs, often results in many cancer-related deaths [5].

Tobacco-related cancers cause premature death among Indian men, causing loss of family income and productivity [6]. In India, an estimated 30%–60% of cancer cases in men and 10%–30% in women are caused by tobacco [7]. Additionally, 80% of tobacco users live in economically developing countries where there is little effort to promote prevention or aid current smokers, many of whom are young [8]. Infections are estimated to be responsible for 20%–25% of all cancer cases worldwide, with a higher incidence in developing countries [9]. In cases of cervical cancers in India, human papillomavirus is detected in 80%–85% of cases, highlighting its role in causing this type of cancer [7]. While early detection can lead to successful treatment, too few women are screened as being at risk [7]. In India, there are 34 million carriers of Hepatitis B, a significantly higher prevalence than in other countries, which increases the risk of developing hepatocellular carcinoma [10]. Additionally, around 2.4 million people in India live with HIV, which puts this population at risk of developing HIV-associated cancers [10].

This narrative review aimed to explore the impact of the growing burden of cancer in India and the palliative and end-of-life (EOL) care strategies initiated to mitigate them.

## Methods

This narrative review was conducted according to the four-step method described by Demiris *et al* [11]. The search was conducted in two multidisciplinary databases, Scopus and Web of Science, using search terms related to the review topic. Further searches were conducted for the individual themes generated to find more evidence. Additional citations, bibliographic and hand search increased the identification of relevant literature. A broad search string palliati* OR hospice OR ‘EOL’ AND cancer OR oncolog* OR malignan* was used. The search was limited to research papers from India, published in English from inception to 31/08/2023. The initial search using the search string limited to India produced 2,341 citations. All titles were screened to identify abstracts and full text relevant to the review topic. Only empirical research was used to describe gaps in palliative care provision. Non-empiric research like editorials, opinion pieces, letters to the editor and grey literature was used to identify palliative and end-of-life (EOL) care initiatives, as empiric research might not capture them comprehensively. The Scale for the Assessment of Narrative Review Articles checklist was used to ascertain the quality of the narrative review [12].

## Results

### Capacity building to bridge gaps in cancer palliative care service provision

In the Indian cancer setting, evidence suggests a prominent gap between palliative care needs and provision. In a survey study with 89 cancer centres participating, there was a gap in the adequacy of palliative care training among oncologists and a lack of palliative care-trained specialists [13]. Moreover, in this study, only two-thirds and one-third of centres had palliative care inpatient and outpatient services, and only two-thirds of the patients receiving cancer-directed therapy had concurrent palliative care consults. Palliative home care facility was accessible to only one-third of the patients. Although most cancer hospitals were licensed to procure and store oral opioids, many patients could not access them. Lack of administrative support, earmarked funds, trained staff and policy hindered palliative care development in cancer centres [13]. Another survey involving 223 palliative care centres in India showed that only 68% provided cancer palliative care, and only 50% provided inpatient palliative care [14]. Uninterrupted opioid access to patients with cancer was a significant challenge [14].

At a national level, a mitigating strategy was to develop palliative care capacity in cancer treatment centres across India through a cancer treatment centre palliative care program (CTC-PC) [15]. In 2023, the CTC-PC is running the 5th edition of its program. In its first three editions, an audit showed that the CTC-PC developed palliative care services in 23 out of 31 centres enrolled in this program [16]. All centres started outpatient services with adequate infrastructure and staff. The availability of morphine also improved, and its use increased from before, indicating better access to opioids. These centres continued education, training and advocacy efforts, which showed that healthcare providers remained committed to the program after completing their training cycle [16].

Another national initiative was the Enable Quality and Improve Patient Care in India program, an international initiative aiming to improve the quality of care for patients with cancer and palliative care needs [17]. The program has now become a leader in supporting national collaborations and training in quality improvement throughout India and is integrated into the National Cancer Grid. Promoting Access and Improvement of Cancer Experience Program (PAICE) has also demonstrated a successful model of international collaboration and capacity building in palliative care and cancer quality improvement. Throughout three cohorts from 2017 to 2020, PAICE provided capacity-building support to 22 Indian palliative care and cancer programs [17].

The lack of palliative care specialists in the cancer centre was a gap, and starting specialist palliative care training was an initiative to bridge this gap. The regulatory authorities recognised Palliative Medicine as a medical subspecialty in 2010. In India, the Specialist Palliative Medicine training program is a supervised 3-year training program offered at a medical college department or a tertiary hospital. The first specialist Palliative Medicine training program began as MD in 2012 at Tata Memorial Hospital in Mumbai. It has led to many Palliative Medicine Departments across India starting MD Palliative Medicine programs. Additionally, in 2021, the National Board of Examinations approved a Diplomate in National Board (DNB) in Palliative Medicine; currently, around ten institutes each offer MD and DNB Palliative Medicine Programs [18].

Furthermore, gaps in palliative care training for oncologists were bridged through several short education programs, both face-to-face and online. In 2007, the Indian Association of Palliative Care (IAPC) established the Certificate Course in Essentials of Palliative Care. Subsequently, the Indo-American Cancer Association launched a 6-week certificate program in palliative care. The EPEC-India program is a joint effort between Indian Palliative Care Providers and Northwestern University of Chicago to provide generalist palliative care education throughout India. The Extension for Community Health Outcome (ECHO) program, an innovative virtual education program, was developed across different regions of India in collaboration with ECHO International [19].

Limited palliative care access in the community and homes, primarily in rural India, is a gap. Assessment of urban community palliative care needs in Delhi, India, showed that 150 per 100,000 population needed palliative care [20]. The need estimated in this survey is significantly lower than that shown in the Global Atlas of Palliative Care for the low–middle-income country, calculated as 993 per 100,000 population [21]. Moreover, in the Delhi study [20], cancer only contributed to 12.5% of palliative care needs, whereas malignant neoplasms contributed to 30% of palliative care needs globally [21]. A qualitative study on palliative care delivery in rural India showed challenges in service provision [22]. These include limited access to palliative care until the later stages of illness, patients being uninformed about their cancer stage, unaffordability of medication and treatment costs, transportation challenges in accessing care and strict regulations on morphine distribution, making it difficult for patients to acquire the drug. Moreover, cultural factors discouraging patients from seeking palliative care and reluctance from the medical community to utilise rural medical practitioners for care were also barriers [22].

There is empirical evidence to suggest that home-based palliative care initiatives are helpful for people with advanced cancers. A study conducted in Mumbai, India, showed that a nurse-coordinated home care service reduced symptoms, emergency health care access, caregiver burden and cost of caring [23]. Another 6-year study of home-based palliative care services in Mumbai, India, found that patients experienced better symptom control, improved quality of life and increased satisfaction among caregivers during the care process [24]. Families expressed satisfaction with the grief support they received from the team [24]. A review of home-based palliative care services in north-eastern India showed that these improved symptoms, access to medications and overall quality of life [25].

Hospitals and non-governmental organisations have initiated community palliative care networks, services and home-based programs across India to overcome this gap. In 2000, the Neighbourhood Network in Palliative Care program was launched to establish a community-led, sustainable service that could offer palliative care to everyone requiring it. This initiative triggered significant social mobilisation, which was feasible in the socially responsible State of Kerala. With the assistance of the PPCS, community-owned units were created in rural regions [26]. Can-Support, Karunashraya, Pal Care, Institute of Palliative Medicine, Pallium, and several other organisations provide exemplary home-based palliative care services across India.

Apart from home-based palliative care services, hospices for patients with advanced cancer have seen steady growth over the last four decades. During the early 1980s, Dr. D’Souza founded a hospice in Mumbai [27]. 1992, the Cipla Cancer and AIDS Foundation created a unique living palliative care centre. Subsequently, Jivodaya Hospice, Pain and Palliative Care Society, and Word and Deed Hospital began providing hospice services in Kerala. In 1994, the Indian Cancer Society, a public charitable trust, partnered with Rotary Bangalore Indiranagar to establish a hospice called Karunashraya in Bangalore. It paved the way for developing several hospices across India [28].

Lack of access to opioids significantly hinders cancer palliative care provision. Opioid access is greatly restricted even in cancer centres, challenging cancer pain management [29]. Moreover, a retrospective audit showed that potent opioids were initiated in only 16% of patients with advanced cancer in a tertiary cancer centre [30]. Limited access to opioids significantly contributes to health-related suffering in South Asian countries [31]. To address this gap, in 2014, the Indian Parliament amended the NDPS Act. It was possible due to the tireless advocacy of the palliative care community. The amendment created a new category called ‘Essential Narcotic Drugs’ (ENDs), which transferred the power of legislating ENDs from the states to the central government, simplifying the procedure. Now, every state and union territory follows a single system, and the drugs controller of the state is the sole authority for approving Recognised Medical Institutions to stock and dispense ENDs. This change facilitated the improvement of the availability of opioids in India. It was primarily due to the collaboration between the government, the palliative care community and the manufacturers of opioids and narcotics [32].

The other constraint in cancer palliative care provision was the lack of organisational support and policies. The Kerala Government was the first state in a low or middle-income country to introduce a palliative care policy in 2008, revised and updated in 2019 [33]. The Indian Government launched the National Program in Palliative Care in 2012 [34], allowing state governments to apply for funding for palliative care projects through program implementation plans. Palliative care was also included in the National Health Policy of 2017, which provided training modules and videos for primary-level implementation. Other states in India have also introduced their palliative care policies besides Kerala [34].

Apart from national policies, specific policies were developed to facilitate EOL care through society position statements, guidance documents and institutional procedural guidelines. The IAPC collaborated with the Indian Society of Critical Care Medicine and the Indian Academy of Neurology to form the End-of-Life Care in India Taskforce. This task force was dedicated to establishing an ethical and legal framework to ensure quality EOL care. After India was ranked poorly in the Lien Foundation’s quality of death report, an EOL care consortium was formed to promote and develop EOL care in the country. As part of this effort, a position statement and policy guidelines were created [35], and the consortium held multiple meetings with the National Accreditation Board of Hospitals (NABH) to bring critical changes to the NABH manual regarding EOL care. The Indian Council of Medical Research (ICMR) also formed an expert group to standardise EOL resuscitation terms and protocols. It resulted in a booklet and publication of 25 defined terms by the ICMR in 2018 [36], and a protocol for limitation of treatment was published in 2020 [37]. Continued advocacy efforts led to a landmark Supreme Court decision in 2023, making EOL care provisions less ambiguous and more accessible [38].

### Strategies to identify and mitigate health-related suffering

According to the 2018 Lancet Commission Report, 7.2 million Indians out of the 1.35 billion population experience health-related severe suffering [39]. A study exploring the health-related suffering experienced by women with cervical cancer showed that a high percentage suffered from moderate to severe pain, vaginal discharge and vaginal bleeding [40]. Additionally, one-third of participants experienced a loss of faith. The study also found that half of the assessed patients experienced clinically significant anxiety and depression. Furthermore, over 80% of these women reported sexual dysfunction, and in many patients, their intimate partners abandoned them [40]. Essential Palliative Care for Cervical Cancer (EPPCCC) was one of the interventions developed to help ease the suffering of patients with cervical cancer [41]. The EPPCCC included various interventions like medicines, essential equipment, social support and human resources, all aimed at preventing and relieving suffering associated with cervical cancer. The package only used affordable and easily accessible medicines and equipment, which only required basic training [41].

Another study conducted in five Asian countries, including India, showed that people residing in households with lower economic status and fewer years of education reported more significant levels of health-related suffering across various domains [42]. The study also showed notable interaction effects between the economic status of the household and education level concerning all EOL suffering outcomes.

Furthermore, age was pivotal in moderating the relationship between the household’s economic status and social suffering and between education level and psychological, social and spiritual suffering [42].

The Lancet Commission on Global Access to Palliative Care and Pain Relief found that health-related suffering is prevalent worldwide, especially in low- and middle-income countries [43]. A screening tool for serious health-related suffering was developed for individual patients in India’s healthcare settings to address this. The instrument consisted of a two-part questionnaire. The first part evaluated and scored the physical, emotional, social, spiritual and financial aspects of health-related suffering. The second part determined the severity of the suffering by assessing functional limitations and the patient’s preferences [43].

Approximately 28 million nurses worldwide comprise 59% of the healthcare workforce [44]. They provide up to 90% of primary health services. To alleviate health-related suffering, nursing leadership development should be promoted as an intervention to increase universal access to palliative care services [44]. The End-of-Life Care Nursing Education Consortium (ELNEC) is an international group of nurses working to enhance nursing education and improve knowledge and attitudes towards palliative and EOL care [45]. In India, a study was conducted using a pre-and post-training questionnaire to assess the impact of ELNEC training on nursing staff. The results showed a significant improvement in knowledge of palliative care and more positive attitudes towards caring for dying patients after the training [45].

India’s multi-profession palliative care education to ease health-related suffering is not limited to doctors and nurses. The IAPC developed several education initiatives, like a palliative pharmacy certificate course and a certificate course in palliative care for volunteers, physiotherapists and counsellors. Other organisations like Karunashraya Institute of Palliative Care Education and Research and Trivandrum Institute of Palliative Sciences offer similar courses.

Health technology development has also had its place in palliative care capacity development in India. A qualitative study on the use of mobile applications for palliative care delivery found that such apps are highly accepted and used frequently [46]. The study also showed that using the apps improved the palliative care team’s understanding of patient symptoms and concerns. However, there is a need for better feedback to caregivers, prioritisation of patients according to their needs, enhanced training and support for app usage and user-led recommendations for ongoing improvement [46]. A tele-triage of palliative care needs using telemedicine during the COVID period also enabled palliative care access and bettered outcomes [47].

### Timely palliative care engagement to improve symptoms and quality of life

Evidence from India suggests that adults and children with cancer received disease-directed therapy until the last weeks of life, and despite symptoms, their palliative care referral was deferred [48–53]. Less than half of the patients were referred to palliative care in an Indian childhood cancer setting, and the median number of days between referral and death was 14 days [54]. An audit of patients with gynaecological malignancies in India revealed pain as the most common symptom, followed by anorexia, constipation and fatigue [49]. Younger patients tended to have a higher symptom burden [49]. Another study conducted in a tertiary cancer centre in India showed that 94% of patients with oesophageal cancer had physical symptoms like pain dysphagia and fatigue [55]. 82% experienced emotional concerns, and 58% had diminished spiritual well-being [55]. A study assessing life problems in patients with advanced cancer in India showed that the majority had difficulties in activities of daily living and performing moderate to heavy work [56]. Most had fatigue and sleep disturbances and feared physical suffering [56]. A qualitative study conducted in Southern India hospitals showed that oncologists’ non-disclosure of cancer diagnosis and prognosis, excessive focus on pain and physical symptoms with non-consideration of psychosocial and spiritual concerns and wanting palliative care to be a pain management service often limited comprehensive palliative care development [57].

Although no established integrated palliative care models exist in India, some evidence supports this emerging trend. A mixed-methods study conducted in a tertiary hospital in India showed that oncologists were unaware of the scope of palliative care and perceived its absence [58]. In a qualitative study, Indian paediatric oncologists suggested several strategies to facilitate an integrated palliative care model [59]. Another research conducted in India demonstrated that by educating oncologists during interdisciplinary meetings, spreading awareness to clinicians and patient families through pamphlets, establishing referral guidelines and screening processes, including symptom burden charts in head and neck cancer clinic notes, and regularly seeking feedback from oncologists during review meetings, the time it took to refer patients to palliative medicine decreased significantly [60]. The average referral time decreased from 48 to 13 days within 6 months following the intervention [60].

A feasibility study on palliative care integration in patients with lung cancer in India showed improved pain and anxiety scores in the first 2 weeks and quality of life in the subsequent visits [61]. A study conducted in a private cancer centre in India on the benefits of palliative care engagement showed that oncologists, oncology nurses and patients all noticed improved symptom control and communication [62]. Oncologists and nurses were particularly pleased with the better management of EOL care, such as clear communication about prognosis, discussions about the limits of life-sustaining treatment and effective symptom management. Patients were happy with the improved continuity of care, including discharge planning, access to necessary medications, follow-up plans and after-hours telephone support [62]. In contrast, two studies from India did not show the benefit of early palliative care engagement in a cancer setting. A randomised controlled trial in Head and Neck Cancer patients showed that palliative care intervention did not improve quality of life [63]. In another study, consultation with a specialist palliative care team did not improve the quality of life in patients with poor performance status [64]. Both these studies were conducted in busy outpatient settings of a tertiary cancer centre, limiting the effectiveness of palliative care intervention.

### Reducing the distress associated with high health care costs

Patients with cancer and their families in India experience distress due to significant therapy-related costs. Evidence suggests that 37% to 49% of a household’s monthly consumption expenditure is spent on inpatient and outpatient cancer care [65]. The cost of cancer hospitalisation in India is generally high, and payment inequities are influenced by income status and the availability of government or private insurance [66]. The increased financial burden of cancer is due to the cost of cancer-directed therapies, prolonged hospital stays and frequent outpatient visits [67].

Due to the low gross domestic product spent on health in India, cancer treatment costs remain high, leading to high out-of-pocket expenses [68]. In a study conducted in Lucknow, India, the out-of-pocket expenditure per patient per day was Indian Rupees 2,044.21 [69]. Most families in India bear the direct and indirect costs of cancer treatment themselves, which often leads to debt due to healthcare expenses [65]. A study in patients with head and neck cancer showed that healthcare system factors, such as the limited availability of comprehensive and meaningful insurance and reimbursements, can intensify the negative effects of treatment [70]. Moreover, the financial burden can significantly harm a patient’s mental and social well-being [70]. An Indian study showed that the prevalence of depression in patients with cancer is as high as 70% [71]. It was associated with residing in a nuclear family, lacking finances and adequate insurance coverage [71]. Moreover, a systematic review examining the impact of cancer care expenses on patients in India found that several factors, including income, type of healthcare facility, disease stage, location, age, recurrence, education, insurance and treatment method, influence the ability to manage treatment costs [72].

Patients accessing treatment in private hospitals pay three times higher costs than a public hospital. They often have to borrow money, sell belongings or rely on family and friends for financial support. Additionally, over 60% of people accessing care in private hospitals in India pay more than 20% of their household income towards cancer treatment [68]. Financial toxicity can lead to a lower quality of life, debt, early employment and difficulty adhering to treatment. These findings highlight the importance of implementing immediate plans to alleviate financial toxicity for cancer patients in India [72].

In India, most hospices provide palliative and EOL care at no cost to patients and families. Similarly, most home-based palliative care services are also offered free of charge. The hospice and home-based palliative care services work on a philanthropic model, funded by individual donors, trusts or big companies as part of their corporate social responsibility. In a hospital setting, the IAPC has advocated with the Government and has developed packages to provide highly subsidised hospital-based palliative care through the Ayushman Bharath scheme [73]. Moreover, steps are taken to induce palliative care as part of the Indian private health insurance through negotiations with private insurance companies.

### Unburdening the heavy-laden caregivers

The disability-adjusted life years (DALYs), a measure of the overall disease burden for cancer in India, was 26.7 million in 2021, projected to increase to 29.8 million in 2025 [3]. Among men in India, DALYs were commonly associated with lung, head, neck and oesophageal cancers, whereas in women, cervical, breast and ovarian cancers contributed to higher DALYs [74]. DALYs are proportional to the caregiving needs. A Zarit Burden Interview that assessed the impact on caregiving burden quality of life (QOL) using the WHO BREF QOL questionnaire showed that 70.22% of Indian caregivers in a cancer setting reported mild-to-moderate burden, whereas 21.38% reported moderate-to-severe burden [75]. In India, cancer is associated with a high caregiving burden, seen in both urban and rural settings, male gender and middle socioeconomic status [76]. These findings mirrored another study from India where family caregivers who were men, never married, unemployed and living in a rural setting in India had a higher caregiving burden, and palliative care access positively impacted caregiving [77]. Furthermore, older caregivers with fewer years in school and past psychological issues or psychiatric diagnoses were associated with negative caregiving experiences [78]. Lack of preparedness, empowerment and availability of people to share caregiving responsibility often increases caregiving burden among Indian women family caregivers [79]. A higher carer burden was also associated with a lack of education and training in caregiving, relentless pursuit of curative options and limited understanding of palliative care scope [80].

The respite model of palliative care was an initiative developed to ease caregiver burden and empower them [81]. Although respite services were designed to give a break to carers from the caregiving task, in India, it has a slightly different connotation. It primarily caters to managing symptoms and complex situations outside a hospital setting. It empowers families to care for patients at home and serves as a transition from hospital to home or hospice [81]. In Mumbai, India, the respite palliative care service had a team of professionals, including physicians, nurses, medical social workers, clinical psychologists, rehabilitation specialists and volunteers. The main goal of this service was to provide a respite for caregivers, offer psychosocial support and counselling and help with economic and vocational rehabilitation. The social workers and trained volunteers assisted patients and their families in managing their symptoms and improve their quality of life [82]. They also facilitated continuity of care by creating a liaison system with family physicians and local palliative care networks. Additionally, they provided after-hour telephonic support and helped patients and their families plan for the future. The ultimate aim was to empower families and caregivers to maintain continuity of care once the patient is discharged from the respite service [82].

## Conclusion

Our review highlights several palliative and EOL care initiatives in India for patients with cancer and their families. Initiatives such as establishing palliative care units in cancer treatment centres, structured palliative care training for developing specialists, improving access to opioids, creating hospices and community palliative care services and developing policies have been beneficial. A new initiative involves creating a screening tool to identify health-related suffering proactively. There are also efforts in cancer centres to develop and test the effectiveness of integrated palliative care for various cancer subsites, though these initiatives are still in the early stages. Efforts to reduce health-related costs have helped patients to manage financial toxicity. Caregivers are supported through some respite care facilities developed across India.

## Competing/Conflicts of interest

None.

## Funding

None.

## Consent for publication

Not applicable.

## Ethical approval

Ethical approval is not needed as information is sourced from published literature in the public domain.

## Availability of data and material

Not applicable.

## Author contribution

NS, KR and AD designed the review. NS, KR and AD developed the search strategy, searched the literature, extracted and synthesised the data and prepared the initial manuscript. It underwent review, editing and approval by SB and SNS, who provided critical input and clarification. Finally, all authors read and approved the final manuscript.
